# To Boys Who Smoke

**Published:** 1893-02

**Authors:** 


### TO BOYS WHO SMOKE.

If boys who smoke would only be sensible and see the folly of it, how much better it would be for them and others ! Can you not see, do you not know—that you are going through a great deal of misery to do something you do not really like ? You are enduring, with a patience worthy of a better cause, the sufferings of a martyr, in order to acquire a useless, bad habit; and trying to cultivate a taste that makes you sick. Why should you treat yourself so meanly? You know perfectly well that you do not smoke because you enjoy it. It is only when you think some one (but assuredly not your parents) is looking at you. You always do this with an air of intense self-consciousness. Every body, including yourselves, know that you are on exhibition. And it is such a pitiable, cheap show, too. You think people are admiring you, which they are not. Why, so far from exciting admiration in the minds of the beholders, if you boys could hear the remarks which people make when they see you smoking, you would never again try a cigarette where human eye could perceive you. Moreover, it makes you disagreeable company. When you bring into society the horrid taint of stale tobacco in your hair and clothes, your absence is always more gratefully welcome than your presence. So don't smoke boys. It makes you stupid, so it does not help you in your studies. It is injurious for the heart, so it does not aid you in athletic sports. It does not do you one particle of good; it makes you appear silly and ridiculous; it is as disagreeable and offensive to yourselves as it is to any body else; you do not get a bit of comfort and real pleasure out of it, and you all know it; so pray do not smoke!

